# Carbapenem resistance, inappropriate empiric treatment and outcomes among patients hospitalized with Enterobacteriaceae urinary tract infection, pneumonia and sepsis

**DOI:** 10.1186/s12879-017-2383-z

**Published:** 2017-04-17

**Authors:** Marya D. Zilberberg, Brian H. Nathanson, Kate Sulham, Weihong Fan, Andrew F. Shorr

**Affiliations:** 1 EviMed Research Group, LLC, PO Box 303, Goshen, MA 01032 USA; 2 OptiStatim, LLC, PO Box 60844, Longmeadow, MA 01116 USA; 3 The Medicines Company, 8 Sylvan Way, Parsippany, NJ 07054 USA; 4 Washington Hospital Center, 110 Irving St. NW, Washington, DC 20010 USA

**Keywords:** UTI, Pneumonia, Sepsis, Enterobacteriaceae, Antimicrobial resistance, Inappropriate empiric therapy, Hospital mortality, Hospital cost

## Abstract

**Background:**

Drug resistance among gram-negative pathogens is a risk factor for inappropriate empiric treatment (IET), which in turn increases the risk for mortality. We explored the impact of carbapenem-resistant Enterobacteriaceae (CRE) on the risk of IET and of IET on outcomes in patients with Enterobacteriaceae infections.

**Methods:**

We conducted a retrospective cohort study in Premier Perspective database (2009–2013) of 175 US hospitals. We included all adult patients with community-onset culture-positive urinary tract infection (UTI), pneumonia, or sepsis as a principal diagnosis, or as a secondary diagnosis in the setting of respiratory failure, treated with antibiotics within 2 days of admission. We employed regression modeling to compute adjusted association of presence of CRE with risk of receiving IET, and of IET on hospital mortality, length of stay (LOS) and costs.

**Results:**

Among 40,137 patients presenting to the hospital with an Enterobacteriaceae UTI, pneumonia or sepsis, 1227 (3.1%) were CRE. In both groups, the majority of the cases were UTI (51.4% CRE and 54.3% non-CRE). Those with CRE were younger (66.6+/−15.3 vs. 69.1+/−15.9 years, *p* < 0.001), and more likely to be African-American (19.7% vs. 14.0%, *p* < 0.001) than those with non-CRE. Both chronic (Charlson score 2.0+/−2.0 vs. 1.9+/−2.1, *p* = 0.009) and acute (by day 2: ICU 56.3% vs. 30.4%, *p* < 0.001, and mechanical ventilation 35.8% vs. 11.7%, *p* < 0.001) illness burdens were higher among CRE than non-CRE subjects, respectively. CRE patients were 3× more likely to receive IET than non-CRE (46.5% vs. 11.8%, *p* < 0.001). In a regression model CRE was a strong predictor of receiving IET (adjusted relative risk ratio 3.95, 95% confidence interval 3.5 to 4.5, *p* < 0.001). In turn, IET was associated with an adjusted rise in mortality of 12% (95% confidence interval 3% to 23%), and an excess of 5.2 days (95% confidence interval 4.8, 5.6, *p* < 0.001) LOS and $10,312 (95% confidence interval $9497, $11,126, *p* < 0.001) in costs.

**Conclusions:**

In this large US database, the prevalence of CRE among patients with Enterobacteriaceae UTI, pneumonia or sepsis was comparable to other national estimates. Infection with CRE was associated with a four-fold increased risk of receiving IET, which in turn increased mortality, LOS and costs.

## Background

Initial antibiotic therapy affects outcomes in severe infection. For empiric therapy to have a benefit on patient outcomes, it must not only be given in a timely manner but must also be active in vitro against the infecting pathogen. Many studies indicate that either delaying antibiotic therapy or selecting a treatment to which the infecting pathogen is non-susceptible increases the risk for death 2–5-fold [1–13]. Therefore, clinicians must be aware of the common pathogens in specific infectious syndromes and of local antimicrobial susceptibility patterns in order to make appropriate choices for antimicrobial therapies. Unfortunately, rapidly rising rates of resistance and shifting resistance patterns render ensuring appropriate empiric coverage a challenge [14].

Recently, the Centers for Disease Control and Prevention have identified carbapenem-resistance among Enterobacteriaceae as an urgent threat in the US [15]. Though Enterobacteriaceae are common pathogens in pneumonia, urinary tract infections and sepsis and thus are often treated in most empiric coverage recommendations, the escalating frequency of carbapenem resistance in these pathogens makes ensuring initially appropriate antimicrobial treatment in areas where carbapenem-resistant Enterobacteriaceae (CRE) are prevalent nearly impossible [13, 14, 16–19]. Furthermore, administering broad-spectrum agents to all severely ill patients in order not to miss some individual with a rare highly resistant pathogen is not a sustainable practice, since the concerns for promoting further resistance may outweigh any potential benefit to patient-specific outcomes. In this way, the dilemma of CREs amplifies the tension between public (preservation of antimicrobial activity) and patient-level (optimizing clinical outcomes) health imperatives.

It remains unclear if the nexus between inappropriate therapy and outcomes seen with other pathogens exists in the case of infections due to CRE. Few analyses have specifically addressed this issue, while some that have attempted this lacked the ability to delineate the impact of inappropriate empiric therapy of CREs on attributable morbidity or on resources such as length of stay (LOS) [20, 21]. To understand better the relationship between carbapenem-resistance, choice of inappropriate empiric therapy (IET), and key hospital outcomes, we conducted a cohort study of patients admitted to the hospital with community-onset urinary tract infections (UTI), pneumonia and sepsis due to Enterobacteriaceae.

## Methods

This was a multi-center retrospective cohort study of patients admitted to the hospital with pneumonia, sepsis and UTI (referred to from here on as “UTI”), or sepsis from another source in the Premier Research database in the years 2009–2013. We hypothesized that infection with a CRE phenotype increased the risk of receiving IET. In turn, we hypothesized that the receipt of IET is adversely associated with hospital mortality, LOS, and costs.

Because this study used already existing fully de-identified retrospective data, it was exempt from IRB review.

Since the data source was the same and methods utilized in this study were similar to those used in our previous study, please refer to that paper for details [22].

### Patient population

Patients were included if they were adults (age ≥ 18 years) hospitalized with a UTI International Classification of Diseases, version 9, Clinical Modification (ICD-9-CM) codes (principal diagnosis 112.2, 590.1, 590.11, 590.2, 590.3, 590.8.590.81, 595, 597, 599 or 996.64, or principal sepsis diagnosis [see below] with UTI as a secondary diagnosis), pneumonia ICD-9-CM codes (principal diagnosis 481–486, or respiratory failure codes [518.81 or 518.84] with pneumonia as a secondary diagnosis) or sepsis codes from another source (principal diagnosis 038, 038.9, 020.0, 790.7, 995.92 or 785.52, or respiratory failure codes [518.81 or 518.84] with sepsis coded as a secondary diagnosis) [23–27]. In order to eliminate confounding of the outcomes by pre-infection onset hospital events, only patients with infection present on admission, as evidenced by antibiotic treatment beginning within the first 2 days of hospitalization and continuing for at least 3 consecutive days, or until discharge, were included [24–26]. Patients were excluded if they were transferred from another acute care facility, if they were diagnosed with cystic fibrosis, or if their hospital length of stay (LOS) was 1 day or less. Those who met criteria for both UTI and sepsis or pneumonia and sepsis were included in the UTI or pneumonia group, respectively. Those with both UTI and pneumonia were analyzed with the pneumonia group. Patients were followed until death in or discharge from the hospital.

### Data source

The data for the study derived from Premier Research database, an electronic laboratory, pharmacy and billing data repository, for years 2009 through 2013, which contains approximately 15% of all hospitalizations nationwide. For detailed description of the dataset, please, refer to citation #22.

### Baseline variables

We classified each community-onset infection (UTI, pneumonia or sepsis) as healthcare-associated (HCA) if one or more of the following risk factors was present: 1) prior hospitalization within 90 days of the index hospitalization, 2) hemodialysis, 3) admission from a long-term care facility, 4) immune suppression [3, 6, 16, 23–26]. All other infections were considered to be community-acquired (CA). For other patient factors and hospital-level variables, please see citation #22.

### Microbiology and treatment variables and definitions

Urinary, blood and respiratory cultures had to be obtained within the first 2 days of hospitalization.

The following organisms were defined as Enterobacteriaceae of interest:

1. *Escherichia coli*
2. *Klebsiella pneumoniae*
3. *Klebsiella oxytoca*
4. *Enterobacter cloacae*
5. *Enterobacter aerogenes*
6. *Proteus mirabilis*
7. *Proteus spp.*
8. *Serratia marcescens*
9. *Citrobacter freundii*
10. *Morganella morganii*
11. *Providencia spp.*

Premier database receives organism susceptibility reports from individual institutions’ laboratories as S (susceptible), I (intermediate) or R (resistant). Although no MIC data are available in the database, all microbiology testing was performed locally at the institutions contributing the data and conformed to the CLSI standards. Carbapenem-resistant Enterobacteriaceae were defined as one of the above organisms where susceptibility testing yielded an I or R result to at least one of the four carbapenems: imipenem, meropenem, ertapenem or doripenem.

IET was present if the antibiotic administered for the infection did not cover the organism or if appropriate coverage did not start within 2 days of the positive culture being obtained.

### Statistical analyses

We compared characteristics of patients infected with CRE to those infected with carbapenem-susceptible Enterobacteriaceae (CSE) and those treated with IET to those treated with non-IET. All unadjusted comparisons were done using standard methods described in detail in citation #22.

We developed a generalized logistic regression model to explore the relationship between CRE and the risk of IET. Covariates in the model were identical to those in citation #22. We calculated the relative risk ratio with 95% confidence intervals of receiving IET for CRE vs. CSE based on Huber-White robust standard errors clustered at the hospital level [28]. Consistent with our prior study, we confirmed our results in a non-parse model and a propensity matched model with propensity for CRE derived from a logistic regression model using the non-parse model’s predictors [22]. To explore the impact of IET on hospital mortality, LOS and costs, we developed hierarchical regression models with hospitals as random effects along with confirmatory propensity-matched models.

All tests were two-tailed, and a *p* value <0.05 was deemed a priori to represent statistical significance. All analyses were performed in Stata/MP 13.1 for Windows (StataCorp LP, College Station, TX).

## Results

Among 230,086 patients presenting to the hospital with a UTI, pneumonia or sepsis, 40,137 (17.4%) met the inclusion criteria for Enterobacteriaceae of which the majority were UTI (54.2%), with the remainder either pneumonia (13.1%) or sepsis (32.7%). Among all patients with Enterobacteriaceae, 1227 (3.1%) had 1938 CRE organisms (Table 1). The prevalence of CRE among the Enterobacteriaceae ranged from 2.9% in UTI to 3.6% in pneumonia. Notably, over 85% of patients in both the CRE and CSE groups had a sepsis diagnosis code at some point during the hospitalization.

**Table 1:** Individual CRE organisms and their frequencies

CRE organism name	CRE organism Count	% of Total CRE	% of the Total patients^a^
	(*N* = 1938)	(*N* = 1938)	(*N* = 1227)
*Klebsiella pneumoniae*	724	37.4%	59.0%
*Proteus mirabilis*	370	19.1%	30.2%
*Escherishia coli*	294	15.2%	24.0%
*Enterobacter cloacae*	128	6.6%	10.4%
*Providencia spp*	94	4.9%	7.7%
*Serratia marcescens*	87	4.5%	7.1%
*Morganella morganii*	87	4.5%	7.1%
*Enterobacter aerogenes*	40	2.1%	3.3%
*Proteus spp.*	27	1.4%	2.2%
*Citrobacter freundii*	27	1.4%	2.2%
*Klebsiella oxytoca*	22	1.1%	1.8%
*Enterobacter other*	13	0.7%	1.1%
*Citrobacter other*	14	0.7%	1.1%
*Serratia other*	6	0.3%	0.5%
*Klebsiella other*	5	0.3%	0.4%

^a^Sum adds up to >100%, as some patients had >1 CRE organism

Those with CRE were younger (66.6+/−15.3 vs. 69.1+/−15.9 years, *p* < 0.001), and more likely to be African-American (19.7% vs. 14.0%, *p* < 0.001) than those with CSE. Many of the individual chronic conditions were more prevalent in the CRE than CSE group, and the mean Charlson comorbidity index reflected this (2.0+/−2.0 vs. 1.9+/−2.1, *p* = 0.009) (Table 2). CRE was more common than CSE in the West and the Northeast, in urban hospitals, in those of medium size (200–499 beds) and in teaching hospitals (*p* < 0.001 for each comparison) (Table 2). Large hospitals (500+ beds) were less likely to have CRE than CSE (Table 2).

**Table 2:** Baseline characteristics

	CSE	%	CRE	%	*P*-value
	*N* = 38,910		*N* = 1227		
Mean age, years (SD)	69.1 (15.9)		66.6 (15.3)		<0.001
Gender: male	16,273	41.8%	642	52.3%	<0.001
Race					
White	28,295	72.7%	821	66.9%	<0.001
Black	5464	14.0%	242	19.7%	
Hispanic	1069	2.7%	32	2.6%	
Other	4082	10.5%	132	10.8%	
Admission Source					
Non-healthcare facility (including from home)	25,559	65.7%	776	63.2%	<0.001
Clinic	1285	3.3%	27	2.2%	
Transfer from ECF	3697	9.5%	266	21.7%	
Transfer from another non-acute care facility	473	1.2%	22	1.8%	
Emergency Department	7766	20.0%	132	10.8%	
Other	130	0.3%	4	0.3%	
Elixhauser Comorbidities					
Congestive heart failure	9623	24.7%	329	26.8%	0.096
Valvular disease	3112	8.0%	96	7.8%	0.825
Pulmonary circulation disease	2323	6.0%	93	7.6%	0.020
Peripheral vascular disease	4285	11.0%	169	13.8%	0.002
Paralysis	4085	10.5%	271	22.1%	<0.001
Other neurological disorders	8668	22.3%	348	28.4%	<0.001
Chronic pulmonary disease	11,035	28.4%	371	30.2%	0.151
Diabetes without chronic complications	11,616	29.9%	420	34.2%	0.001
Diabetes with chronic complications	3809	9.8%	141	11.5%	0.049
Hypothyroidism	6764	17.4%	224	18.3%	0.428
Renal failure	10,810	27.8%	446	36.3%	<0.001
Liver disease	2084	5.4%	65	5.3%	0.929
Peptic ulcer disease with bleeding	17	0.0%	1	0.1%	0.428
AIDS	12	0.0%	0	0.0%	1.000
Lymphoma	604	1.6%	21	1.7%	0.657
Metastatic cancer	1787	4.6%	40	3.3%	0.027
Solid tumor without metastasis	1569	4.0%	34	2.8%	0.026
Rheumatoid arthritis/collagen vascular	1721	4.4%	45	3.7%	0.204
Coagulopathy	5350	13.7%	139	11.3%	0.015
Obesity	6095	15.7%	191	15.6%	0.926
Weight loss	6855	17.6%	340	27.7%	<0.001
Fluid and electrolyte disorders	21,332	54.8%	378	30.8%	0.764
Chronic blood loss anemia	545	1.4%	24	2.0%	0.105
Deficiency anemia	15,154	38.9%	598	48.7%	<0.001
Alcohol abuse	1367	3.5%	33	2.7%	0.122
Drug abuse	923	2.4%	35	2.9%	0.278
Psychosis	2358	6.1%	81	6.6%	0.435
Depression	5854	15.0%	174	14.2%	0.404
Hypertension	24,938	64.1%	781	63.7%	0.752
Charlson Comoribidity Score					
0	12,010	30.9%	334	27.2%	<0.001
1	7855	20.2%	230	18.7%	
2	7902	20.3%	244	19.9%	
3	5118	13.2%	180	14.7%	
4	2897	7.4%	146	11.9%	
5+	3128	8.0%	93	7.6%	
Mean (SD)	1.9 (2.1)		2.0 (2.0)		0.009
Median [IQR]	1 [0,3]		2 [0, 3]		<0.001
Hospital Characteristics					
Census region					
Midwest	10,531	27.1%	288	23.5%	<0.001
Northeast	5297	13.6%	336	27.4%	
South	16,203	41.6%	310	25.3%	
West	6879	17.7%	293	23.9%	
Number of Beds					
< 200	6589	16.9%	192	15.6%	<0.001
200 to 299	8779	22.6%	338	27.5%	
300 to 499	12,691	32.6%	421	34.3%	
500+	10,851	27.9%	276	22.5%	
Teaching	14,609	37.5%	566	46.1%	<0.001
Urban	35,079	90.2%	1167	95.1%	<0.001

*CSE* carbapenem sensitive Enterobacteriaceae, *CRE* carbapenem resistant Enterobacteriaceae, *SD* standard deviation, *ECF* extended care facility, *AIDS* acquired immune deficiency syndrome, *IQR* interquartile range

In both the CRE and CSE groups, over one-half the patients had the diagnosis of UTI, with the remaining divided between sepsis (33.3% CRE vs. 32.7% CSE) and pneumonia (15.2% CRE vs. 13.0% CSE) (Table 2). Patients infected with CRE were more likely to have a HCA infection (58.5% vs. 35.4%, *p* < 0.001) along with a greater illness severity by day 2 of admission (ICU 56.0% vs. 40.8%, *p* < 0.001; mechanical ventilation 35.6% vs. 15.7%, *p* < 0.001; though not vasopressors 16.7% vs. 14.9%, *p* = 0.081) than CSE patients (Table 3). Although among the CRE group there was a higher prevalence of empiric use of carbapenems, aminoglycosides and polymyxins than in those eventually found to be infected with a CSE, those with CRE infections were also significantly more likely to receive IET (52.8% vs. 11.1%, *p* < 0.001). Unadjusted hospital mortality median LOS and costs among CRE were also significantly greater than CSE, and these differences held across all infection types (Table 3).

**Table 3:** Infection characteristics, treatment and outcomes

	CSE	%	CRE	%	*P*-value
	*N* = 38,910		*N* = 1227		
Infection characteristics					
Sepsis	12,726	32.7%	409	33.3%	0.039
Pneumonia	5060	13.0%	187	15.2%	
UTI	21,124	54.3%	631	51.4%	
HCA	13,782	35.4%	718	58.5%	<0.001
Illness severity measures by day 2					
ICU admission	15,876	40.8%	687	56.0%	<0.001
Mechanical ventilation	6092	15.7%	437	35.6%	<0.001
Vasopressors	5798	14.9%	205	16.7%	0.081
Antibiotics administered by day 2					
Aminoglycosides	3843	9.9%	242	19.7%	<0.001
Antipseudomonal penicillins	6403	16.5%	313	25.5%	<0.001
Antipseudomonal floroquinolones	18,468	47.5%	406	33.1%	<0.001
Antipseudomonal penicillins with beta-lactamase inhibitors	19,727	50.7%	617	50.3%	0.775
Extended spectrum cephalosporins	13,327	34.3%	415	33.8%	0.755
Folate pathway inhibitors	251	0.6%	12	1.0%	0.155
Penicillins with beta-lactamase inhibitors	854	2.2%	26	2.1%	0.837
Polymyxins	126	0.3%	24	2.0%	<0.001
Tetracyclines	248	0.6%	6	0.5%	0.519
Tigecycline	586	1.5%	86	7.0%	<0.001
Aztreonam	1740	4.5%	56	4.6%	0.878
Empiric treatment appropriateness					
Non-IET	32,197	82.7%	513	41.8%	<0.001
IET	4336	11.1%	648	52.8%	
Indeterminate	2337	6.0%	66	5.4%	
*Hospital outcomes*					
Mortality	3958	10.2%	178	14.5%	<0.001
Mean (SD) LOS, days	9.6 (10.7)		15.6 (17.4)		<0.001
Median [IQR] LOS, days	7 [4, 11]		10 [6, 18]		<0.001
Mean (SD) costs, $	20,601 (29702)		38,494 (46,964)		<0.001
Median [IQR] costs, $	13,020 [7501, 24,237]		22,909 [12,988, 42,815]		<0.001
*Hospital outcomes stratified by infection type*					
UTI					
Mortality	1873	8.9%	78	12.4%	0.002
Mean (SD) LOS, days	9.0 (9.4)		14.6 (15.9)		<0.001
Median [IQR] LOS, days	7 [4, 11]		10 [6, 17]		<0.001
Mean (SD) costs, $	19,036 (24,494)		33,400 (37,662)		<0.001
Median [IQR] costs, $	12,082 [7104, 21,822]		21,154 [12,687, 39,374]		<0.001
Sepsis					
Mortality	1660	13.0%	81	19.8%	<0.001
Mean (SD) LOS, days	10.9 (12.6)		18.0 (20.8)		<0.001
Median [IQR] LOS, days	7 [4, 13]		11 [7, 21]		<0.001
Mean (SD) costs, $	26,793 (37,390)		50,038 (60,602)		<0.001
Median [IQR] costs, $	15,614 [8584, 30,317]		27,264 [14,581, 57,825]		<0.001
Pneumonia					
Mortality	425	8.4%	19	10.2%	0.395
Mean (SD) LOS, days	9.2 (10.4)		13.4 (13.0)		<0.001
Median [IQR] LOS, days	7 [4, 10]		9 [6, 16]		<0.001
Mean (SD) costs, $	19,250 (25,743)		30,432 (35,089)		<0.001
Median [IQR] costs, $	11,826 [7076, 21,100]		19,820 [12,220, 35,713]		<0.001

*CSE* carbapenem sensitive Enterobacteriaceae, *CRE* carbapenem resistant Enterobacteriaceae, *UTI* urinary tract infection, *HCA* healthcare-associated, *ICU* intensive care unit, *IET* inappropriate empiric therapy

Comparing the cohort of 37,694 patients (93.9% of all patients with Enterobacteriaceae) with valid antimicrobial treatment data, 32,710 (86.8%) received appropriate therapy (Table 4). While patients receiving appropriate empiric therapy were more likely to have UTI or sepsis than those in the IET group, the frequency of pneumonia was higher among patients on IET (20.0%) than those on appropriate treatment (12.0%) (*p* < 0.001) (Table 4). As for the unadjusted hospital outcomes, mortality was higher in patients receiving IET than appropriate therapy (12.2% vs. 9.9%, *p* < 0.001). Both LOS and costs were significantly higher in the IET group than in the group receiving non-IET (Table 4). These relationships generally held irrespective of the infection type (Table 4).

**Table 4:** Characteristics of the cohort based on the receipt of inappropriate empiric treatment

	Non-IET	%	IET	%	*P*-value
	*N* = 32,710		*N* = 4984		
*Baseline characteristics*					
Mean age, years (SD)	69.0 (16.0)		69.4 (15.3)		0.094
Gender: male	13,680	41.8%	2169	43.5%	0.024
Race					
White	23,921	73.1%	3443	69.1%	<0.001
Black	4384	13.4%	862	17.3%	
Hispanic	919	2.8%	163	3.3%	
Other	3486	10.7%	516	10.4%	
Admission Source					
Non-healthcare facility (including from home)	21,450	65.6%	3034	60.9%	<0.001
Clinic	1093	3.3%	138	2.8%	
Transfer from ECF	2996	9.2%	759	15.2%	
Transfer from another non-acute care facility	379	1.2%	77	1.5%	
Emergency Department	6688	20.4%	959	19.2%	
Other	104	0.3%	17	0.3%	
Elixhauser Comorbidities					
Congestive heart failure	7836	24.0%	1509	30.3%	<0.001
Valvular disease	2594	7.9%	425	8.5%	0.148
Pulmonary circulation disease	1912	5.8%	358	7.2%	<0.001
Peripheral vascular disease	3564	10.9%	577	11.6%	0.152
Paralysis	3289	10.1%	770	15.4%	<0.001
Other neurological disorders	7227	22.1%	1269	25.5%	<0.001
Chronic pulmonary disease	9079	27.8%	1663	33.4%	<0.001
Diabetes without chronic complications	9695	29.6%	1623	32.6%	<0.001
Diabetes with chronic complications	3152	9.6%	524	10.5%	0.052
Hypothyroidism	5645	17.3%	942	18.9%	0.004
Renal failure	9024	27.6%	1540	30.9%	<0.001
Liver disease	1774	5.4%	245	4.9%	0.138
Peptic ulcer disease with bleeding	15	0.0%	2	0.0%	1.000
AIDS	8	0.0%	4	0.1%	0.063
Lymphoma	508	1.6%	74	1.5%	0.716
Metastatic cancer	1543	4.7%	182	3.7%	0.001
Solid tumor without metastasis	1335	4.1%	163	3.3%	0.006
Rheumatoid arthritis/collagen vascular	1422	4.3%	215	4.3%	0.914
Coagulopathy	4626	14.1%	540	10.8%	<0.001
Obesity	5079	15.5%	822	16.5%	0.081
Weight loss	5583	17.1%	1117	22.4%	<0.001
Fluid and electrolyte disorders	17,961	54.9%	2702	54.2%	0.357
Chronic blood loss anemia	459	1.4%	79	1.6%	0.313
Deficiency Anemia	12,735	38.9%	2096	42.1%	<0.001
Alcohol abuse	1139	3.5%	163	3.3%	0.446
Drug abuse	789	2.4%	103	2.1%	0.135
Psychosis	1979	6.1%	294	5.9%	0.676
Depression	4859	14.9%	806	16.2%	0.018
Hypertension	20,987	64.2%	3154	63.3%	0.229
Charlson Comoribidity Score					
0	10,353	31.7%	1239	24.9%	<0.001
1	6517	19.9%	1072	21.5%	
2	6595	20.2%	1047	21.0%	
3	4223	12.9%	757	15.2%	
4	2400	7.3%	465	9.3%	
5+	2622	8.0%	404	8.1%	
Mean (SD)	1.9 (2.1)		2.0 (2.0)		<0.001
Median [IQR]	1 [0, 3]		2 [1 3]		<0.001
*Infection characteristics and treatment*					
Infection characteristics					
Sepsis	10,736	32.8%	1468	29.5%	<0.001
Pneumonia	3936	12.0%	995	20.0%	
UTI	18,038	55.1%	2521	50.6%	
HCA	11,413	34.9%	2221	44.6%	<0.001
CRE	513	1.6%	648	13.0%	<0.001
Illness severity					
ICU admission	13,524	41.3%	2074	41.6%	0.720
Mechanical ventilation	5064	15.5%	1062	21.3%	<0.001
Vasopressors	4929	15.1%	709	14.2%	0.111
Antibiotics administered					
Aminoglycosides	3694	11.3%	351	7.0%	<0.001
Antipseudomonal penicillins	6199	19.0%	347	7.0%	<0.001
Antipseudomonal floroquinolones	15,995	48.9%	2480	49.8%	0.258
Antipseudomonal penicillins with beta-lactamase inhibitors	16,874	51.6%	2008	40.3%	<0.001
Extended spectrum cephalosporins	12,174	37.2%	1134	22.8%	<0.001
Folate pathway inhibitors	225	0.7%	36	0.7%	0.809
Penicillins with beta-lacatamase inhibitors	681	2.1%	147	2.9%	0.005
Polymyxins	102	0.3%	32	0.6%	<0.001
Tetracyclines	210	0.6%	15	0.3%	0.004
Tigecycline	485	1.5%	110	2.2%	<0.001
Aztreonam	1319	4.0%	258	5.2%	<0.001
*Hospital Characteristics*					
Area					
Midwest	8848	27.0%	1133	22.7%	<0.001
Northeast	4397	13.4%	950	19.1%	
South	13,579	41.5%	1951	39.1%	
West	5886	18.0%	950	19.1%	
Number of Beds					
< 200	5597	17.1%	744	14.9%	<0.001
200 to 299	7508	23.0%	1171	23.5%	
300 to 499	10,540	32.2%	1781	35.7%	
500+	9065	27.7%	1288	25.8%	
Teaching	12,096	37.0%	1988	39.9%	0.217
Urban	29,418	89.9%	4574	91.8%	<0.001
*Hospital outcomes*					
Mortality	3234	9.9%	607	12.2%	<0.001
Mean (SD) LOS, days	9.0 (8.5)		14.7 (19.4)		<0.001
Median [IQR] LOS, days	7 [4, 11]		9 [5, 16]		<0.001
Mean (SD) costs, $	20,227 (25,616)		33,216 (49,567)		<0.001
Median [IQR] costs, $	12,719 [7401, 23,275]		17,386 [9255, 35,625]		<0.001
*Hospital outcomes stratified by infection type*					
UTI					
Mortality	1548	8.6%	267	10.6%	<0.001
Mean (SD) LOS, days	8.5 (7.8)		13.3 (17.1)		<0.001
Median [IQR] LOS, days	6 [4, 10]		9 [5, 15]		<0.001
Mean (SD) costs, $	18,103 (21,440)		28,069 (40,490)		<0.001
Median [IQR] costs, $	11,862 [7015, 21,222]		16,209 [8828, 31,535]		<0.001
Sepsis					
Mortality	1356	12.6%	260	17.7%	<0.001
Mean (SD) LOS, days	9.9 (9.9)		18.9 (23.3)		<0.001
Median [IQR] LOS, days	7 [4, 12]		12 [6, 22]		<0.001
Mean (SD) costs, $	24,532 (32,043)		47,881 (64,812)		<0.001
Median [IQR] costs, $	15,048 [8312, 28,558]		25,121 [12,382, 55,529]		<0.001
Pneumonia					
Mortality	330	8.4%	80	8.0%	0.726
Mean (SD) LOS, days	8.5 (7.6)		12.0 (17.6)		<0.001
Median [IQR] LOS, days	7 [4, 10]		7 [4, 13]		<0.001
Mean (SD) costs, $	18,220 (21,710)		24,623 (38,753)		<0.001
Median [IQR] costs, $	11,742 [7125, 20,561]		13,040 [7393, 26,339]		<0.001

*IET* inappropriate empiric therapy, *SD* standard deviation, *ECF* extended care facility, *AIDS* acquired immune deficiency syndrome, *IQR* interquartile range, *HCA* healthcare-associated, *CSE* carbapenem sensitive Enterobacteriaceae, *CRE* carbapenem resistant Enterobacteriaceae, *UTI* urinary tract infection, *ICU* intensive care unit, *IQR* interquartile range 25–75%

In a parse generalized regression model exploring the impact of CRE on the risk of IET, resistance was the single strongest predictor of receiving IET (adjusted relative risk ratio 3.95, 95% confidence interval 3.51, 4.46, *p* < 0.001) (Table 5). The confirmatory analyses produced similar risk ratios (Table 5).

**Table 5:** Adjusted risk of inappropriate empiric therapy, hospital mortality, excess LOS and costs

	Marginal effect, CSE	Marginal effect, CRE	Adjusted relative risk ratio/excess days or costs (95% confidence interval)	*P*-value
*Risk of IET*				
Parse Model	11.8%	47.7%	3.95 (3.51, 4.46)	<0.001
Propensity score (based on 100% CRE cases matched to CSE 1:1)	13.1%	55.8%	4.27 (3.64, 5.00)	<0.001
Non-parse model	11.9%	47.7%	4.00 (3.48, 4.59)	<0.001
	Marginal effect, non-IET	Marginal effect, IET	Adjusted relative risk ratio/excess days or costs (95% confidence interval)	*P*-value
*Risk of death*				
Hierarchical model	9.8%	11.0%	1.12 (1.03, 1.23)	0.013
Propensity score (based on 96.4% IET cases matched to non-IET 1:1)	10.5%	11.9%	1.13 (1.01, 1.27)	0.030
*Length of stay (days)*				
Hierarchical model	8.2	13.4	5.2 (4.8, 5.6)	<0.001
Propensity score (based on 96.4% IET cases matched to non-IET 1:1)	9.6	14.6	5.0 (4.4, 5.6)	<0.001
*Hospital costs*				
Hierarchical model	$20,508	$30,819	$10,312 ($9497, $11,126)	<0.001
Propensity score (based on 96.4% IET cases matched to non-IET 1:1)	$22,005	$32,837	$10,831 ($9254, $12,409)	<0.001

*IET* inappropriate empiric therapy, *CSE* carbapenem sensitive Enterobacteriaceae, *CRE* carbapenem resistant Enterobacteriaceae

In a hierarchical regression model adjusting for all confounders (demographics, comorbidities, severity of illness measures, hospital characteristics) IET was associated with an increased risk of in-hospital mortality (adjusted relative risk ratio 1.12; 95% confidence interval 1.03, 1.23, *p* = 0.013) (Table 5). In other hierarchical models, the excess LOS and costs associated with IET exposure were 5.2 days (95% confidence interval 4.8, 5.6, *p* < 0.001) and $10,312 (95% confidence interval $9497, $11,126, *p* < 0.001). Propensity-matched analyses produced similar estimates (Table 5).

An interaction term suggested a greater impact on mortality of IET in the setting of sepsis, which prompted a sensitivity analysis in the group whose organisms were cultured from blood. In this set of analyses, including 12,807 patients (186 CRE, 1.5%), the impact of IET on mortality was indeed greater (relative risk ratio 1.55, 95% confidence interval 1.18 to 2.03) than in the overall cohort.

## Discussion

We demonstrate in this large multicenter observational cohort that among patients admitted from the community with a UTI, sepsis or pneumonia, over 17% have an infection with Enterobacteriaceae, of which approximately 3% are CRE. Although infrequent, the presence of CRE increases the risk of receiving IET substantially. In turn, receiving IET is associated with a rise in hospital mortality, LOS and costs, a rise particularly pronounced in patients with sepsis.

Multiple studies have noted an increase in the prevalence of CRE among patients with serious infections in the hospital. A recent US surveillance study reported the annual population incidence of CRE infections to be nearly 3 cases per 100,000 population [28]. A US Centers for Disease Control and Prevention study noted a rise in CRE prevalence from 1.2% in 2001 to 4.2% in 2011 [29]. The same study analyzing a different database, however, noted an increase in CRE from 0 in 2001 to 1.4% in 2010, echoing findings of other investigators [19, 30, 31]. Our findings are generally in agreement with these numbers. Although CRE incidence and prevalence are far lower than such common pathogens as methicillin-resistant *Staphylococcus aureus* or *Clostridium difficile*, there are few treatment alternatives for CRE, which underscores the need for more precise information about the epidemiology and outcomes related to CRE infections [32, 33]. Consequently, this study helps to address this need for more granular information regarding this pathogen. In addition we confirm that at this point, CRE is encountered most often as a urinary pathogen, which may mediate the otherwise high mortality rate associated with CRE infections. Despite this, the increasing frequency of this organism as a cause of sepsis indicates that CRE is poised to become a major contributor to infectious disease related mortality in the US.

Though thought of mostly as healthcare-associated pathogens, our data suggest that this may be too narrow a view. Namely, in our cohort, over 40% of patients with CRE did not have an identifiable exposure to the healthcare system. There are several potential sources for misclassifying this burden, one of which may be the 90-day period for prior hospitalization as a risk factor for HCA infection. Though it remains unclear how long the impact of prior hospitalization persists on the risk of resistance, and 90 days is a standard interval used in many other studies, in some investigations this period is longer [34]. Although a probable overestimate due to misclassification and because of limitations in the patient records, our data are not the first to bring into question this assumption in a US population. In the surveillance study of CRE by Guh et al., 2/3 of the cultures derived from the outpatient setting [35]. More importantly, 8% lacked any markers of healthcare exposure [35]. In an additional small study by Tang et al., community-acquired CRE accounted for 30% of all CRE infections [36]. Though higher in our study, the fact remains that persons with no ongoing relevant exposure to the healthcare system may still contract an infection with this organism. This finding is troubling in that it parallels what has been observed with extended-spectrum beta-lactamase carrying pathogens and their increasing prevalence in community-acquired infections [37–40].

There is mounting evidence to demonstrate that rising antimicrobial resistance impedes clinical efforts at instituting appropriate empiric treatment [14]. We confirm the important role resistance plays in thwarting the ability to choose appropriately, whereby the risk of receiving IET in the setting of CRE rose 4-fold compared to CSE. In turn, though modest, IET’s adverse impact on hospital mortality is consistent with what has been reported in other infections [1–13]. The more pronounced impact of IET on hospital LOS (~5 excess days) and costs (~additional $10,000) is a novel finding for infections with CRE, and provides a sound rationale for investing in technologies that identify patients at risk for CRE more rapidly, particularly given that this is approximately double the attributable burden reported in infections caused by other resistant organisms [41]. Moreover, having a precise estimate of the attributable costs of these infections helps put into perspective the potential value of various prevention and treatment paradigms. It is methodologically challenging to estimate the attributable impact of carbapenem resistance on cost and LOS in nosocomial CRE infections since those outcomes are confounded by the cause of the initial hospitalization. Therefore, our findings help clarify this issue.

Our study has a number of strengths and limitations. The limitations that are common to both the current and previous studies are discussed in citation #22. Specific to the current analysis, a potential source of misclassification is a relatively high prevalence of *Proteus mirabilis* as a pathogen, as this microbe may have naturally occurring higher MICs for imipenem (Table 1) [42, 43]. Since susceptibility data in Premier are reported not by the MIC, but by susceptibility designation (S, I, R, see above in Methods), for the purpose of this analysis we had to presume that clinical adjudication occurred at each individual institution. However, this type of misclassification, if present, is likely to lead to an underestimate of the impact of CRE on outcomes, thus suggesting that in fact, CRE, when determined without this potential misclassification, may have an even greater effect on the risk of IET exposure.

## Conclusions

In summary, CRE is an uncommon but important pathogen in community-onset UTI, pneumonia and sepsis. We confirm that, similar to other resistant organisms, it evades appropriate empiric treatment and exposure to IET worsens both clinical and economic outcomes. Although the true extent of the problem requires further study, our data confirm that a substantial proportion of CRE may be acquired in the community irrespective of exposure to the healthcare system. In sum, our study provides compelling evidence to hasten development of rapid identification methods and new antibiotic treatments in order to optimize empiric therapy among hospitalized patients with serious infections.

## Abbreviations

CA: Community-acquired
CRE: Carbapenem-resistant Enterobaceriaceae
CSE: Carbapenem-sensitive Enterobacteriaceae
HCA: Healthcare-associated
ICU: Intensive care unit
IET: Inappropriate empiric therapy
LOS: Length of stay
UTI: Urinary tract infection
